# A Differential Effect of Lovastatin versus Simvastatin in Neurodevelopmental Disorders

**DOI:** 10.1523/ENEURO.0162-20.2020

**Published:** 2020-07-28

**Authors:** Melania Muscas, Sang S. Seo, Susana R. Louros, Emily K. Osterweil

**Affiliations:** 1 Centre for Discovery Brain Sciences, University of Edinburgh, Edinburgh, EH8 9XD, United Kingdom; 2 Simons Initiative for the Developing Brain, University of Edinburgh, Edinburgh, EH8 9XD, United Kingdom

**Keywords:** autism, ERK, fmr1, fragile X, lovastatin

## Significance Statement

The statin drug lovastatin normalizes excessive protein synthesis and thereby ameliorates pathologic changes in animal models of fragile X syndrome (FX), the most commonly identified genetic cause of autism. Recently, we compared the efficacy of lovastatin to the more potent and brain-penetrant drug simvastatin for correcting phenotypes in the *Fmr1^-/y^* mouse (Muscas et al., 2019). Surprisingly, we find simvastatin worsens excessive protein synthesis and has no impact on audiogenic seizures (AGS) in *Fmr1^-/y^* mice, suggesting it does not work in a similar fashion to lovastatin. A recent commentary by Ottenhoff et al. (2020) suggests that differences in dose and/or study design might account for our results. Here, we discuss the points raised by Ottenhoff et al. as well as the evidence supporting a therapeutic role for lovastatin versus simvastatin. We conclude that differences between lovastatin and simvastatin warrant careful consideration with respect to the treatment of neurodevelopmental disorders.

## 

Therapeutic strategies that reduce protein synthesis have shown efficacy in reducing pathologic brain phenotypes in fragile X syndrome (FX; Stoppel et al., 2017; Protic et al., 2019). In the FX (*Fmr1^-/y^*) mouse model, lovastatin reduces the activation of Ras and downstream extracellular regulated-kinase (ERK) signaling, thereby normalizing protein synthesis and correcting changes in synaptic plasticity, neuronal hyperexcitability, epileptogenesis, and learning (Osterweil et al., 2013; Sidorov et al., 2014; Table 1). In the *Fmr1^-/y^* rat model, early administration of lovastatin prevents emergence of plasticity deficits and learning deficiencies later in development (Asiminas et al., 2019). In recent work, we tested whether the structurally similar drug simvastatin could correct core phenotypes of excessive hippocampal protein synthesis and audiogenic seizures (AGS) in the *Fmr1^-/y^* mouse (Muscas et al., 2019). The motivation for testing simvastatin versus lovastatin is a two- to four-fold increase in potency, increased brain penetrance, and wider availability in Europe (Schachter, 2005). However, simvastatin has not been tested in any model of FX, and preclinical evidence of efficacy was required before incurring the significant cost of a clinical trial. This is particularly relevant for simvastatin, which has been tested for the treatment of neurofibromatosis type 1 (NF1), a neurodevelopmental disorder characterized by excessive Ras-ERK signaling. Early studies in the *Nf1*+/− mouse showed a significant correction of several brain phenotypes with lovastatin (Li et al., 2005). Assuming the mechanisms for reversing pathologic changes were identical for lovastatin and simvastatin, clinical trials were initiated for simvastatin in NF1 despite the absence of animal model studies. To date, three randomized placebo-controlled clinical trials for simvastatin in NF1 have failed to show a significant improvement in primary outcome measures (Krab et al., 2008; van der Vaart et al., 2013; Stivaros et al., 2018; Table 2).

**Table 1:** Animal model studies of lovastatin and simvastatin in neurodevelopmental disorders

Lovastatin				
Model	Dose	Administration	Effect on phenotype	Reference
*Fmr1^-/y^* mouse	10–100 μm 30–100 mg/kg 10 mg/kg/d	Bath application Injection i.p. Oral feeding 2 d	Rescue: excessive protein synthesis Exaggerated plasticity (mGluR-LTD) Epileptogenesis (hippocampal slice) Hyperexcitability (visual cortical slice) AGS	Osterweil et al. (2013)
*Fmr1^-/y^* mouse	10 mg/kg/d	Oral feeding 2 weeks	Rescue: visuospatial learning No rescue: exaggerated extinction of visuospatial learning	Sidorov et al. (2014)
*Fmr1^-/y^* mouse	50 μm 100 mg/kg	Bath application Injection i.p.	Rescue: excessive protein synthesis AGS	Muscas et al. (2019)
*Fmr1^-/y^* mouse	50 μm	Bath application	No rescue: hyperexcitability and altered gamma (visual cortical slice)	Goswami et al. (2019)
*Fmr1^-/y^* rat	10 mg/kg/d	Oral feeding 2 weeks	Rescue: excessive protein synthesis Plasticity deficits (LTP PFC slice), learning impairments	Asiminas et al. (2019)
*Ube3a^m-/p+^* mouse	50–100 μm 10–100 mg/kg	Bath application Injection i.p.	Rescue: hyperexcitability (hippocampal slice) AGS	Chung et al. (2018)
*Nf1^+/-^* mouse	10 mg/kg/d	Injection i.p. or oral feeding	Rescue: hyperactive ERK signaling Plasticity deficit (LTP hippocampal slice) Attention deficit Impaired spatial learning (MWM) Impaired sensory gating (PPI)	Li et al. (2005)
*Mecp2^-/y^* mouse	1.5 mg/kg	Injection s.c. twice weekly	Rescue: impaired locomotor activity	Buchovecky et al. (2013)
*Ptpn11^D61G/+^* mouse	10 mg/kg	Injection s.c.	Rescue: excessive Ras-ERK in brain Deficient LTP Impaired spatial learning (MWM)	Buchovecky et al. (2013)
*En2^-/-^* mouse	10 mg/kg/d	Injection s.c.	Rescue: hyperactive ERK signaling No rescue: impaired spatial learning (MWM)	Provenzano et al. (2014)
Simvastatin				
Model	Dose	Administration	Effect on phenotype	Reference
*Fmr1^-/y^* mouse	3–50 mg/kg 0.1–5 μm	Injection i.p. Bath application	No rescue: AGS Worsening: Excessive protein synthesis	Muscas et al. (2019)

Studies using animal models of neurodevelopmental disorders have tested the impact of lovastatin on multiple phenotypes. Ours is the only study of simvastatin in a neurodevelopmental animal model.

i.p.: intraperitoneal, s.c.: subcutaneous, mGluR-LTD: metabotropic glutamate receptor stimulated long-term depression, LTP: long-term potentiation, PFC: prefrontal cortex, ERK: extracellular-regulated kinase, MWM: Morris Water Maze, PPI: pre-pulse inhibition.

**Table 2:** Human studies of lovastatin and simvastatin in neurodevelopmental disorders

Lovastatin				
Disorder	Dose	Study type	Results	Reference
FX	Escalating dose 20–40 mg/d 12 weeks	Open-label *N* = 15 6–31 years	Improvement: aberrant behavior [aberrant behavior checklist (ABC), clinical global impression scale (CGI-S), and vineland adaptive behavior scale] Excessive ERK signaling in platelets	Caku et al. (2014); Pellerin et al. (2016)
FX	10–40 mg/d with PILI 12 weeks	RCT with PILI *N* = 28 10–17 years	No improvement: language (standardized tests, parent reported visual analogue scale) Behavior (ABC)	Thurman et al. (2020)
NF1	Escalating dose 20–40 mg/d 3 months	Open-label *N* = 24 10–17 years	Improvement: verbal memory Non-verbal memory Resting state functional connectivity (MRI)	Acosta et al. (2011); Chabernaud et al. (2012)
NF1	200 mg/d 4 d	RCT *N* = 22 19–31 years	Improvement: intracortical inhibition and synaptic plasticity (transcranial magnetic stimulation), alertness (test of attentional performance)	Mainberger et al. (2013)
NF1	40–80 mg/d 14 weeks	RCT *N* = 32 10–50 years	Improvement: working memory Declarative memory Verbal fluency Self-reported internalizing No improvement: neural activity (fMRI) Spatial learning (arena maze)	Bearden et al. (2016); Ullrich et al. (2020)
NF1	Escalating dose 20–40 mg/d 16 weeks	RCT *N* = 146 8–15 years	No improvement: visuospatial learning attention	Payne et al. (2016)

Simvastatin				
Disorder	Dose	Study type	Results	Reference
NF1	Dose escalation 10 to 20–40 mg/d 12 weeks	RCT *N* = 62 8–16 years	No improvement: delayed recall (Rey complex figure test), Attention (cancellation test) Coordinated hand movement (prism adaptation task) Mean brain apparent diffusion coefficient (MRI)	Krab et al. (2008)
NF1	Dose escalation 10 to 20–40 mg/d 12 months	RCT *N* = 82 8–16 years	No improvement: intelligence (Wechsler intelligence scale) Attention (child and parent behavior checklist) Internalizing behaviors (child and parent behavior checklist)	van der Vaart et al. (2013)
NF1	Dose escalation 30 mg/d 12 weeks	RCT *N* = 26 4.5–10.5 years	No improvement: hyperactive ERK in platelets GABA in frontal white matter (MR spectroscopy) Resting state fMRI Aberrant behavior (ABC, CGI-S, parent questionnaire)	Stivaros et al. (2018)
Autism	20–40 mg/d as add on to risperidone (1–2 mg/d) 10 weeks	RCT with riperidone *N* = 70 4–12 years	Improvement: irritability and hyperactivity (ABC)	Moazen-Zadeh et al. (2018)

Lovastatin and simvastatin have been tested in clinical trials for FX and NF1, with varying outcomes.

RCT: randomized placebo-controlled trial; ABC: aberrant behavior checklist, CGI-S: clinical global impression scale, MRI: magnetic resonance imaging, ERK: extracellular-regulated kinase, GABA: γ-Aminobutyric acid.

**Figure 1.**
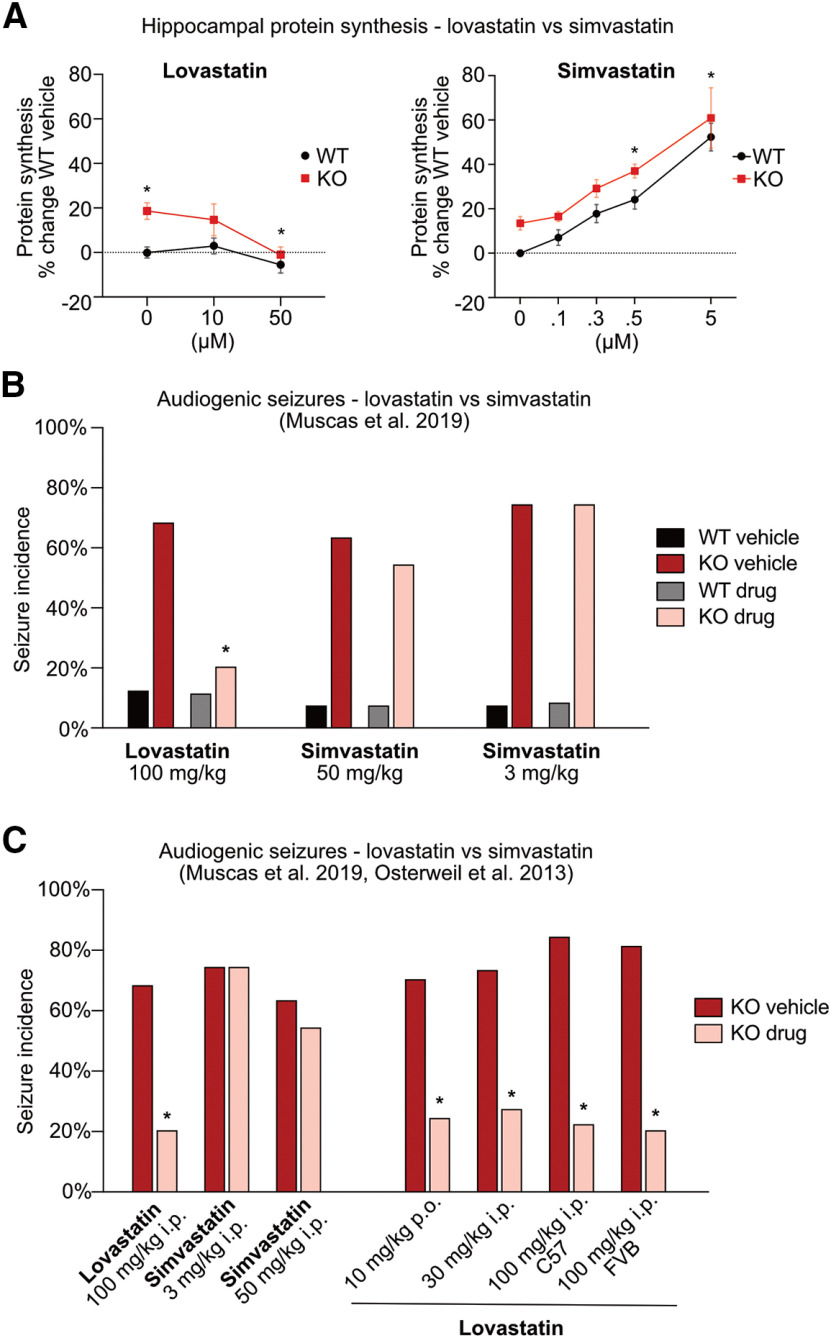
Lovastatin, not simvastatin, corrects fragile X phenotypes. ***A***, Data from Osterweil et al. (2013) and Muscas et al. (2019) were combined and re-analyzed. Metabolic labeling was performed on hippocampal slices prepared from WT/*Fmr1^-/y^* littermates as previously described. A dose-response curve shows lovastatin corrects excess protein synthesis in the *Fmr1^-/y^* hippocampus at 50 μm (two-way repeated measures mixed-model ANOVA treatment *p* = 0.0052, genotype *p* = 0.0006, genotype × treatment *p* = 0.0438; Sidak’s WT veh vs KO veh *p = 0.0021, KO veh vs KO 50 *p = 0.0014). In contrast, simvastatin significantly raises protein synthesis in a dose-dependent manner in both *Fmr1^-/y^* and WT hippocampus (two-way repeated measures mixed-model ANOVA treatment *p* < 0.0001, genotype *p* = 0.0005, genotype × treatment *p* = 0.9754, Sidak’s WT veh vs WT 0.5 *p = 0.0120, WT veh vs WT 5 *p < 0.0001, KO veh vs KO 0.5 *p = 0.0157, KO veh vs KO 5 *p < 0.0001). ***B***, Data re-plotted from Muscas et al. (2019; Extended Data Figure 1-1). AGS assays show that acute injection of 100 mg/kg lovastatin significantly reduces the incidence of seizures in *Fmr1^-/y^* mice versus vehicle control (Fisher’s exact test *p = 0.0136). Conversely, neither an equipotent dose of 50 mg/kg simvastatin (Fisher’s exact test *p* = 0.6968) nor a lower 3 mg/kg dose significantly (Fisher’s exact test *p* > 0.999) impacts the incidence of seizures in the *Fmr1^-/y^* mouse. ***C***, AGS results from Muscas et al. (2019) and Osterweil et al. (2013) show that although simvastatin fails to reduce seizures, lovastatin significantly reduces seizures when given at 10 mg/kg orally for 2 d, 30 mg/kg injection (intraperitoneal), or 100 mg/kg injection (intraperitoneal) in *Fmr1^-/y^* mice on both C57BL6 and FVB background strains (Fisher’s exact test: 10 mg/kg *p = 0.003, 30 mg/kg *p = 0.041, 100 mg/kg C57 *p = 0.005, 100 mg/kg FVB *p = 0.005; Extended Data Figs. 1-2, 1-3).

To our surprise, the comparison of lovastatin and simvastatin in the FX mouse model revealed significant differences. While lovastatin reduces protein synthesis in *Fmr1^-/y^* hippocampus to wild-type (WT) levels, simvastatin resulted in a significant increase in protein synthesis in both genotypes (Fig. 1*A*). In contrast to lovastatin, simvastatin does not reduce ERK activation in *Fmr1^-/y^* hippocampus, which is a key driver of the excess protein synthesis phenotype (Osterweil et al., 2010; Muscas et al., 2019). Moreover, simvastatin does not reduce the incidence of AGS in *Fmr1^-/y^* mice, even when administered at a limiting high dose (Fig. 1*B*). In contrast, lovastatin-treated cohorts show a significant reduction in seizure incidence, consistent with previous work (Fig. 1*C*; Osterweil et al., 2013). From these results, we conclude that lovastatin and simvastatin do not work in a similar fashion with respect to FX models and suggest caution should be used when assuming these compounds are interchangeable. Our results have been discussed in a recent commentary by Ottenhoff et al. (2020), who have been involved in clinical trials with simvastatin for the treatment of NF1 (Krab et al., 2008; van der Vaart et al., 2013; Stivaros et al., 2018; Ottenhoff et al., 2020). The authors raise points regarding our study design, suggesting differences in dose and/or study design might account for the failure of simvastatin to correct *Fmr1^-/y^* phenotypes. Here, we discuss these points and examine the evidence supporting lovastatin versus simvastatin for the treatment of neurodevelopmental disorders.

10.1523/ENEURO.0162-20.2020.f1-1 Extended Data Figure 1-1 Original dataset from Muscas et al. (2019). Download Figure 1-1, XLS file.

10.1523/ENEURO.0162-20.2020.f1-2 Extended Data Figure 1-2 Combined dataset from Muscas et al. (2019) and 100 mg/kg dataset from Osterweil et al. (2013). Download Figure 1-2, XLS file.

10.1523/ENEURO.0162-20.2020.f1-3 Extended Data Figure 1-3 Combined dataset from Muscas et al. (2019) and four datasets from Osterweil et al. (2013). Download Figure 1-3, XLS file.

## Different Actions on Protein Synthesis

Multiple treatments that normalize excess protein synthesis also ameliorate epileptogenic and behavioral phenotypes in FX models (Dölen et al., 2007; Liu et al., 2011; Gkogkas et al., 2014; Gantois et al., 2017; Stoppel et al., 2017). To investigate whether simvastatin corrects the excessive protein synthesis phenotype in the *Fmr1^-/y^* mouse, we used a metabolic labeling assay in hippocampal slices that has been employed in previous studies (Osterweil et al., 2010). As the potency of simvastatin is two- to four-fold that of lovastatin (Schaefer et al., 2004), we chose a starting dose of 5 μm, which is half the 10 μm starting dose of lovastatin used in previous work (Osterweil et al., 2013). Remarkably, this relatively modest dose of simvastatin caused a 50–60% increase in protein synthesis in both WT and *Fmr1^-/y^* slices, dramatically worsening the protein synthesis phenotype (Fig. 1*A*; Muscas et al., 2019). Given these results, we reasoned that increasing concentration would not only be ineffective, it would have deleterious consequences for both WT and *Fmr1^-/y^* hippocampus. Instead, we tested whether a lower dose range of 0.1–0.5 μm simvastatin might mitigate potential off-target effects and reduce the protein synthesis phenotype. Unfortunately, increased protein synthesis continued to be seen in slices treated at these lower doses (Fig. 1*A*). In contrast, WT/*Fmr1^-/y^* littermates treated with 50 μm lovastatin resulted in the expected decrease in protein synthesis in *Fmr1^-/y^* slices.

Looking at these results, it is clear that under conditions where lovastatin normalizes protein synthesis in the *Fmr1^-/y^* hippocampus, simvastatin causes a dramatic worsening of this core phenotype. Regarding these results, Ottenhoff et al. state the following:

“the most surprising finding of the study by Muscas and colleagues is the finding that simvastatin treatment at low dose actually worsened the Fmr1 phenotype by further increasing protein synthesis rates. (…) For the follow-up of these trials it would be of great importance to know if a comparable (low) dose of lovastatin (below the doses needed to inhibit ERK) would have a similar negative effect on this phenotype, especially since the dose that can be safely used in clinical trials is much lower than the in vivo dose used in this study.”

We note that dose-response studies have in fact shown that lovastatin decreases protein synthesis at 1, 10, and 20 μm in cultured neuroblasts (Santa-Catalina et al., 2008). In hippocampal slices, we have established that a lower dose of 10 μm lovastatin does not cause a significant reduction in protein synthesis; however, it certainly does not cause the dramatic increase seen with simvastatin (Fig. 1*A*; Osterweil et al., 2013). In contrast, the impact of simvastatin on protein synthesis in neuronal cells has not been determined. The study cited by Ottenhoff et al. describes experiments performed in a muscle-derived C2C12 cell line, and it is not unreasonable to expect that the response in the nervous system will differ (Tuckow et al., 2011). Indeed, simvastatin has been shown to have a number of brain-specific effects that could contribute to the rise in protein synthesis, including a stimulation of neurotrophin release and augmentation of the expression and activation of NMDA-type glutamate receptors (NMDARs; Parent et al., 2014; Roy et al., 2015; Chen et al., 2016). With respect to the latter, acute application of simvastatin has been shown to enhance surface expression and current flow through NMDARs in hippocampal slices, increasing the magnitude of long-term potentiation (LTP; Parent et al., 2014; Chen et al., 2016). The changes in calcium influx and downstream signaling that are associated with NMDAR activation could contribute to the rise of protein synthesis we observe. In contrast, lovastatin has been shown to downregulate the GluN2B subunit of the NMDAR and thereby reduce associated signaling (Huo et al., 2014). This opposing action on NMDARs may contribute to the differential action on protein synthesis in hippocampal slices.

However, it should be noted that longer treatments with simvastatin, lovastatin, and other statins reduce the production of cholesterol needed to stabilize NMDARs at the cell surface, ultimately causing a mild reduction in activity (Zacco et al., 2003; Ponce et al., 2008; Huo et al., 2014; McFarland et al., 2014). Therefore, longer-term experiments testing protein synthesis at multiple timepoints post simvastatin treatment are needed to determine whether changes in NMDAR activity are involved. What we can conclude for now is that the differential impact of lovastatin and simvastatin on basal protein synthesis is striking and should be investigated in follow-up studies.

## Different Actions on ERK

Statins inhibit the 3-hydroxy-3-methylglutaryl coenzyme A (HMG-CoA) reductase pathway that produces both cholesterol and isoprenoid intermediates, which are important substrates for the posttranslational modification and activation of many proteins (Liao, 2005; Ling and Tejada-Simon, 2016; Nürenberg and Volmer, 2012). Lovastatin has been shown to inhibit the Ras farnesylation required for membrane association and subsequent activation of the ERK pathway (Schafer et al., 1989; Mendola and Backer, 1990; Li et al., 2005). In our comparison study, we find that the low doses of simvastatin that raise protein synthesis have no significant impact on ERK activation in the *Fmr1^-/y^* hippocampus (Muscas et al., 2019). Ottenhoff et al. argue that this result conflicts with previous work that shows “like lovastatin, simvastatin has been shown to decrease ERK signaling.” We note that the simvastatin dose used in our study is low because of the impact of higher doses on protein synthesis, and it may be that higher doses of simvastatin ultimately show an inhibitory effect on ERK. However, it is important to consider that the cited studies either do not measure ERK (Guillén et al., 2004; Ghittoni et al., 2006; Ghosh et al., 2009) or show that simvastatin reduces ERK signaling in non-neuronal cells only when Ras-ERK is hyperstimulated, but not under basal conditions (Fürst et al., 2002; Miura et al., 2004; Ghittoni et al., 2005; Khanzada et al., 2006; Ogunwobi and Beales, 2008; Sundararaj et al., 2008; Kang et al., 2009; Chen et al., 2010; Lee et al., 2011; Takayama et al., 2011).

Unlike simvastatin, lovastatin has been shown to reduce basal Ras-ERK signaling in the absence of activation (Santa-Catalina et al., 2008; Osterweil et al., 2013). This point is particularly relevant to the protein synthesis phenotype in FX, which is not because of a hyperactivation of the ERK pathway but rather a hypersensitive response to normal levels of ERK signaling (Osterweil et al., 2010). It is also important to point out that clinical studies of platelets isolated from simvastatin-treated NF1 patients show no significant reduction in basal ERK activation (Stivaros et al., 2018), whereas those isolated from lovastatin-treated FX patients exhibit a robust reduction in ERK signaling that is correlated with treatment efficacy (Pellerin et al., 2016). Future studies examining the mechanistic differences between these statins could be particularly valuable for understanding the impact on neurologic phenotypes.

## Different Actions on AGS

The AGS phenotype has been used to test multiple potential pharmacological strategies that have moved on to clinical investigation in FX, including lovastatin (Yan et al., 2005; Liu et al., 2012; Busquets-Garcia et al., 2013; Osterweil et al., 2013; Gkogkas et al., 2014; Gantois et al., 2017; Stoppel et al., 2017). In Muscas et al. (2019), we compared acute injection of 100 mg/kg lovastatin to an equipotent dose of 50 mg/kg simvastatin. The results show a clear reduction in seizure incidence and severity with lovastatin, and no effect of simvastatin (Fig. 1*B*). Although Ottenhoff et al. argue “there is no experiment in which lovastatin and simvastatin are compared at the same dose (and with the same vehicle),” the differential potency of these drugs has been well established (Schachter, 2005). If the question is whether there is an equivalent impact of these drugs, we would argue equivalent potency is a key point. Moreover, our attempts to increase simvastatin to 100 mg/kg revealed deleterious side effects that would have made it impossible to make a meaningful comparison.

Ottenhoff et al. bring up the important point that “the dose in which a particular drug rescues a phenotype in animal model does not always translate into a clinically applicable and safe dose in humans.” In our study, we compared acute injections of relatively high doses of lovastatin and simvastatin because of the rapid action of these higher doses on the AGS phenotype (Osterweil et al., 2013). However, we also tested a lower dose of 3 mg/kg that is consistent with the dose given to humans according to standard calculations (Nair and Jacob, 2016; Fig. 1*B*). Similar to the higher dose of simvastatin, the 3 mg/kg dose also failed to reduce seizures in the *Fmr1^-/y^* mouse. In contrast, a range of lovastatin doses correct the AGS phenotype in *Fmr1^-/y^* mice including a 2-d 10 mg/kg oral administration that is consistent with a human dose (Fig. 1*C*). This correction of AGS with lovastatin is seen whether *Fmr1^-/y^* mice are bred on the FVB or C57BL6 background strains (Osterweil et al., 2013). Ottenhoff et al. argue “if a behavioral rescue is observed in young mice (e.g., the rescue of seizures in Fmr1 mice was performed on P18-P29 mice; Osterweil et al., 2013; Muscas et al., 2019), it is important to investigate if such a rescue is still observed when the brain has fully matured.” We note that multiple studies in mouse and rat models of FX and other neurodevelopmental disorders have shown that lovastatin corrects pathologic phenotypes over a range of animal ages, including adults (Table 1). In contrast, beyond our study, there is no previous work examining simvastatin in any animal model of neurodevelopmental disorders including the *Nf1*+/− mouse.

## Study Design

From the side-by-side experiments comparing lovastatin versus simvastatin, we conclude there are differences in mechanism and efficacy that should be considered and further investigated in additional animal model studies. Ottenhoff et al. question whether the differences we report are in fact significant, stating “the drugs should not only be tested side-by-side as interleaved experiments, they should also directly be compared with each other using a statistical analysis that tests for a main effect of treatment, and if significant, followed by a post hoc analysis to compare the drugs.” Our experimental design compares lovastatin and simvastatin to matched vehicle groups, rather than directly to one another, because different concentrations of dimethylsulfoxide (DMSO) were needed for each drug. The blinded comparison of drug groups to counter-balanced vehicle controls is considered good practice by multiple authorities on experimental design for laboratory animals (Festing and Altman, 2002).

In order to evaluate the effects of lovastatin and simvastatin on seizure incidence, we used a Fisher’s exact test that allows for comparisons between small (<50) nominal (yes/no) datasets, consistent with previous AGS studies (Pacey et al., 2009; Osterweil et al., 2010, 2013; Henderson et al., 2012; Michalon et al., 2012; Ronesi et al., 2012; Gross et al., 2015; Thomson et al., 2017). We find a significant difference in seizure incidence between vehicle and lovastatin-treated *Fmr1^-/y^* mice (48%, *p* = 0.0136), but not vehicle versuslow-dose simvastatin (0%, *p* > 0.999) or vehicle versushigh-dose simvastatin (9%, *p* = 0.6968; Fig. 1*C*). However, Ottenhoff et al. suggest that fitting our data to a logistic regression model is a better approach for determining global effects of treatment and genotype in all groups. They go on to fit our data to a model and state that it “shows a trend for a main effect of treatment (χ^2^(6)=12; p=0.07), but not for the interaction between genotype and treatment (χ^2^(4)=4; p=0.3). When performing a post-hoc Tukey’s test, neither the Fmr1-lovastatin versus Fmr1 ‘low dose’ of simvastatin (p= 0.96) nor the Fmr1-lovastatin versus Fmr1-‘high dose’ of simvastatin treatment (p>0.99) are significantly different from each other. Hence, despite the fact that the lovastatin dose was 2-30 fold higher than simvastatin dose, it does not seem to perform significantly better than simvastatin in this seizure assay.”

To investigate this issue, we examined the R script used to run the logistic regression model (shared by Ottenhoff et al.). Our analysis revealed a script error that led to the wrong reporting of *p* values from the Tukey’s *post hoc* tests. Running a corrected script shows lower *p* values for the comparisons of lovastatin and simvastatin in *Fmr1^-/y^* mice than originally published (Table 3). Additionally, Ottenhoff et al. run a type 1 ANOVA that assumes an interaction between genotype and treatment, which we do not claim (nor can we with such a low incidence of seizures in WT). Re-running the logistic regression using a type 2 ANOVA that does not assume an interaction shows a trend toward a main effect of treatment, though this does not reach significance (*p* = 0.053). However, our original study was not powered to directly compare treatment groups, and we therefore investigated whether adding an additional treatment group would change the outcome of this analysis. In the original study testing lovastatin in *Fmr1^-/y^* mice, multiple drug doses were tested in both FVB and C57BL6 background strains (Osterweil et al., 2013; Fig. 1*C*). After adding the data from the FVB group treated with 100 mg/kg lovastatin in this study, we re-ran the logistic regression and find a significant effect of treatment (*p* = 0.00021). When both lovastatin groups are collapsed, the significance of this effect increases (*p* = 9.22 × 10 ^−5^). Adding all lovastatin groups from Osterweil et al. (2013) increases the significance further (*p* = 8.08 × 10^−9^; Table 4). Therefore, the logistic regression identifies the difference in treatment when given a dataset of sufficient size. Moreover, we find that a multinominal regression model that examines seizure severity scores reveals a significant treatment effect, even when applied to the original dataset from Muscas et al. (2019; *p* = 0.033; Table 4). The important conclusion is that whether our results are analyzed directly or fit to a more complex model, they show that lovastatin corrects the AGS phenotype in *Fmr1^-/y^* mice, and simvastatin does not.

**Table 3:** Reordered comparisons reveal correct p values for Tukey’s post-hoc tests

Test	Ottenhoff et al. (incorrect order)			Muscas et al. (corrected order)		
	Estimate	*z* value	*p* value	Estimate	*z* value	*p* value
WT, Veh vs lova	0.1542	0.168	1.0000	0.1542	0.168	1.0000
WT, simvalow vs Veh	***–0.4700***	***–0.366***	0.9997	–0.2288	–0.196	1.0000
WT, simvahigh vs Veh	***–0.3830***	***–0.297***	0.9999	–0.3159	–0.271	0.9999
WT, simvalow vs lova	***–0.3159***	***–0.271***	0.9999	–0.3830	–0.297	0.9999
WT, simvahigh vs lova	***–0.2288***	***–0.196***	1.0000	–0.4700	–0.366	0.9997
WT, simvalow vs simvahigh	–0.0870	–0.059	1.0000	–0.0870	–0.059	1.0000
KO, Veh vs lova	–2.1016	–2.872	0.0406	–2.1016	–2.872	0.0406
KO, simvalow vs Veh	***1.4816***	***1.666***	0.5570	0.2963	0.397	0.9995
KO, simvahigh vs Veh	***2.3979***	***2.573***	0.0932	–0.6200	–0.897	0.9607
**KO, simvalow vs lova**	***–0.6200***	***–0.897***	**0.9607**	2.3979	2.573	**0.0932**
**KO, simvahigh vs lova**	***0.2963***	***0.397***	**0.9995**	1.4816	1.666	**0.5570**
KO, simvalow vs simvahigh	–0.9163	–1.017	0.9288	–0.9163	–1.017	0.9288

The regression model R script used by Ottenhoff et al. (2020) assigns different functions to set up the regression model matrix (“unique”) versus the Tukey’s contrast matrix (“tables”). This results in different order of groups for the two matrices, which results in assignment of different headings to the test results. An altered version of the script with the factors level set in the same order for the model matrix and contrast matrix shows the correct Tukey’s test results (see Extended Data Figure 1-3). Estimate and *z* value are multiplied by –1 to reflect the corresponding tests headings. Reversed values are italicized and the corrected *p* values reported by Ottenhoff are in bold.

**Table 4:** Regression model of AGS incidence and severity shows significant treatment effect in lovastatin versus simvastatin groups

Regression model	Genotype effect	Treatment effect	Interaction effect
Logistical regression, type 2 ANOVA (Muscas et al., 2019)	*p* = 6.22 × 10^–12^	*p* = 0.053	*p* = 0.263
Logistical regression (Muscas et al., 2019) + 100 mg/kg lovastatin from Osterweil et al. (2013; lovastatin groups separated)	*p* = 1.58 × 10^–13^	*p* = 0.00021	*p* = 0.4
Logistical regression (Muscas et al., 2019) + 100 mg/kg lovastatin from Osterweil et al. (2013; lovastatin groups collapsed)	*p* = 1.86 × 10^–13^	*p* = 9.22 × 10^–5^	*p* = 0.5
Logistical regression (Muscas et al., 2019) + all lovastatin groups from Osterweil et al. (2013)	*p* < 2.2 × 10^–16^	*p* = 8.08 × 10^–9^	*p* = 0.4
Multinominal regression (Muscas et al., 2019)	*p* = 8.62 × 10^–12^	*p* = 0.033	*p* = 0.34

Re-running the logistical regression comparing lovastatin and simvastatin treatments using a type 2 ANVOA shows a non-significant trend towards an effect of treatment. Adding data from the FVB 100 mg/kg lovastatin group originally published in Osterweil et al. (2013) shows a significant treatment effect either when kept separate or when collapsed into the existing lovastatin group. Adding data from additional lovastatin treatment groups from C57BL6 cohorts from Osterweil et al. (2013; 10, 30, and 100 mg/kg) further increases the significance of the treatment effect. As the interaction of genotype and treatment does not reach significance using this model, it may be that lovastatin corrects seizures in both WT and *Fmr1^-/y^* mice equally; however, the low number of animals have seizures in the WT groups makes this difficult to assess. To compare lovastatin versus simvastatin treatment groups, a multinomial regression model of seizure severity scores with genotype and treatment effect was performed in R using the multinom function in the nnet package (see Extended Data Figure 1-3).

## Future Considerations

Our studies in *Fmr1^-/y^* animal models show promising results for lovastatin that are not seen with simvastatin. However, it is important to note that the role of statins in the treatment of fragile X and other neurodevelopmental disorders will ultimately depend on large scale double-blind placebo-controlled trials. In the case of lovastatin, the results from double-blind placebo-controlled trials for NF1 are mixed, with one showing a significant improvement in verbal and nonverbal memory (Bearden et al., 2016), and another showing no significant effect on visuospatial learning and attention (Payne et al., 2016). In FX, a recent small-scale double-blind trial showed no additional effect of lovastatin on parent implemented language intervention (Thurman et al., 2020). For simvastatin, three randomized placebo controlled clinical trials have failed to show efficacy in NF1 (Table 2). At present, our study represents the only exploration of simvastatin in an animal model of neurodevelopmental disorders. We agree with Ottenhoff et al. that “importance of looking at effective dosing ranges, and more detailed (in vivo) pharmacological studies in animal models should be performed to elucidate the dose-dependency of therapeutic benefit.” Whether simvastatin shows benefits in FX or other models using a specific dosing regimen or alternative behavioral assays is an open question that would be very informative for future clinical studies. What is clear from our initial work is that there are significant differences between the action of lovastatin and simvastatin on brain function that warrant further attention.

10.1523/ENEURO.0162-20.2020.t3-1 Extended Data Table 3-1 R script for logistical regressions. Download Table 3-1, TXT file.

## Synthesis

Reviewing Editor: Alfonso Represa, INSERM Decisions are customarily a result of the Reviewing Editor and the peer reviewers coming together and discussing their recommendations until a consensus is reached. When revisions are invited, a fact-based synthesis statement explaining their decision and outlining what is needed to prepare a revision will be listed below. The following reviewer(s) agreed to reveal their identity: Mark Bear.

Numerous neuropsychiatric symptoms of fragile X syndrome (FX) are believed to be a consequence of altered regulation of protein synthesis at synapses. A number of strategies have been used to restore normal protein synthesis in FX animal models, resulting in amelioration of a variety of mutant phenotypes. These findings have led to the hypothesis that a disease-modifying therapy could be developed for FX based on rebalancing protein synthesis and synaptic function. Yet despite promising results in preclinical studies, to date there is no FDA approved treatment for children with FX and there is therefore an urgent need to discover new treatment strategies. Remarkably an already approved drug lovastatin has been found to reduce the activation of Ras and downstream extracellular regulated-kinase (ERK) signaling and correct aberrant protein synthesis and many other mutant phenotypes in animal models of FX. This is potentially of great importance as lovastatin is widely prescribed, has a known safety profile, and is approved for use in children. Furthermore, a recent study in a rat model of FX showed that early administration of lovastatin prevents emergence of plasticity deficits and learning deficiencies later in development (Asiminas et al., 2019), and has further increased interest in the potential of targeting Ras/ERK signaling with statins in FX patients.

Recent work by the authors testing the efficacy of simvastatin, a more potent and brain penetrant statin, found that it was not as effective as lovastatin in treating mutant phenotypes in the mouse, and urged caution in treating these two drugs as interchangeable. These findings have been commented on by Ottenhoff et al., suggesting differences in dose and/or study design might account for the failure of simvastatin to correct FX phenotypes.

In this rebuttal, authors carefully re-analyzed their data, and compare them to the available literature. The proposed explanations in support of a real different effect of lovastatin and simvastatin., Overall the ideas discussed in the manuscript are compelling and carefully explained. The authors do an excellent job of addressing the major criticisms of Ottenhoff regarding study design and effective dosing ranges. Only a few points require revision.

Minor Points:

1. An understanding of why lovastatin acts oppositely to simvastatin on the protein synthesis phenotype, would seem to be at the very heart of this matter. The authors argue that simvastatin is likely increasing activation of NMDA-type glutamate receptors (NMDAR) and that contributes to the increased protein synthesis in hippocampal slices. Yet it has been reported that some beneficial effects of lovastatin are due to the downregulation of excessive NR2B expression and NMDAR pathway activation (Huo 2014). If the authors are suggesting the increase in protein synthesis by simvastatin is due to increases in NMDAR signaling they should also comment on the possibility that the opposite effect of lovastatin is due to decreasing NMDAR signaling.

2. A long list of statins (rosuvastatin, atorvastatin, mevastatin and pravastatin) including simvastatin has been reported to produce neuroprotective effects via inhibition of NMDAR in other studies (and as mentioned above lovastatin also decreases NMDAR signaling). The authors’ argument that simvastatin-induced increases in NMDAR activation cause increased protein synthesis in hippocampal slices seems to be at odds with the many studies showing neuroprotective effects of simvastatin and other statins following NMDA treatment. The authors could comment on any potential explanations for why (1) simvastatin is having different actions on NMDAR from the other statins and (2) why simvastatin has been reported to have different effects on NMDAR pathway activation in different contexts.

3. Line 94 page 3 “does not a significant” should read “does not cause a significant...”.

4. In Table 1, En2-/- should be writte with 2 NOT superscript.

## Author Response

Dear Alfonso,

Thank you for judging our article potentially suitable for publication. We thank the Reviewers’ for their comments and have incorporated additional text that we believe speaks to the points raised. We feel these revisions have strengthened the manuscript. I have detailed the changes below, and we hope that this will allow us to see the work published in eNeuro. If there are any concerns please let me know.

Sincerely,

Emily Osterweil

Authors’ Rebuttal:

In this rebuttal, authors carefully re-analyzed their data, and compare them to the available literature. The proposed explanations in support of a real different effect of lovastatin and simvastatin., Overall the ideas discussed in the manuscript are compelling and carefully explained. The authors do an excellent job of addressing the major criticisms of Ottenhoff regarding study design and effective dosing ranges. Only a few points require revision.

We thank the reviews for these comments, they are much appreciated.

1. An understanding of why lovastatin acts oppositely to simvastatin on the protein synthesis phenotype, would seem to be at the very heart of this matter. The authors argue that simvastatin is likely increasing activation of NMDA-type glutamate receptors (NMDAR) and that contributes to the increased protein synthesis in hippocampal slices. Yet it has been reported that some beneficial effects of lovastatin are due to the downregulation of excessive NR2B expression and NMDAR pathway activation (Huo 2014). If the authors are suggesting the increase in protein synthesis by simvastatin is due to increases in NMDAR signaling they should also comment on the possibility that the opposite effect of lovastatin is due to decreasing NMDAR signaling.

This is an excellent point, and we agree that the impact of statins on NMDAR activity is a complex issue. Our statement was meant to suggest that changes to NMDAR activity are one way that simvastatin could have brain-specific effects on protein synthesis, however this is speculation.

To clarify we have added the following text:

Indeed, simvastatin has been shown to have a number of brain-specific effects that could contribute to the rise in protein synthesis, including a stimulation of neurotrophin release and augmentation of the expression and activation of NMDA-type glutamate receptors (NMDARs) (Parent et al., 2014; Roy et al., 2015; Chen et al., 2016). With respect to the latter, acute application of simvastatin has been shown to enhance surface expression and current flow through NMDARs in hippocampal slices, increasing the magnitude of long-term potentiation (LTP) (Parent et al., 2014; Chen et al., 2016). The changes in calcium influx and downstream signaling that are associated with NMDAR activation could contribute to the rise of protein synthesis we observe. In contrast, lovastatin has been shown to downregulate the GluN2B subunit of the NMDAR and thereby reduce associated signaling (Huo et al., 2014). This opposing action on NMDARs may contribute to the differential action on protein synthesis in hippocampal slices.

2. A long list of statins (rosuvastatin, atorvastatin, mevastatin and pravastatin) including simvastatin has been reported to produce neuroprotective effects via inhibition of NMDAR in other studies (and as mentioned above lovastatin also decreases NMDAR signaling). The authors’ argument that simvastatin-induced increases in NMDAR activation cause increased protein synthesis in hippocampal slices seems to be at odds with the many studies showing neuroprotective effects of simvastatin and other statins following NMDA treatment. The authors could comment on any potential explanations for why (1) simvastatin is having different actions on NMDAR from the other statins and (2) why simvastatin has been reported to have different effects on NMDAR pathway activation in different contexts.

We agree that the reported stimulation of NMDAR activity by simvastatin and our results showing stimulation of protein synthesis is seemingly at odds with the known role of statins in neuroprotection, and we have added text to point out this disparity. We mention that the difference between acute effects in slice versus longer-term effects seen in vivo should be taken into consideration, and that further studies are needed to determine how simvastatin and lovastatin ultimately act differently on NMDARs.

To clarify we have added the following text:

However, it should be noted that longer treatments with simvastatin, lovastatin and other statins reduce the production of cholesterol needed to stabilize NMDARs at the cell surface, ultimately causing a mild reduction in activity (Zacco et al., 2003; Ponce et al., 2008; Huo et al., 2014; McFarland et al., 2014). Therefore, longer-term experiments testing protein synthesis at multiple timepoints post simvastatin treatment are needed to determine whether changes in NMDAR activity are involved. What we can conclude for now is that the differential impact of lovastatin and simvastatin on basal protein synthesis is striking, and should be investigated in follow up studies.

3. Line 94 page 3 “does not a significant” should read “does not cause a significant...”.

This has been corrected.

4. In Table 1, En2-/- should be writte with 2 NOT superscript.

This has been corrected.
